# Supplementary figures and images for: The clock-like accumulation of germline and somatic mutations can arise from the interplay of DNA damage and repair
Source: PLoS Biol. 2024 Jun 17;22(6):e3002678. doi: 10.1371/journal.pbio.3002678 (PMC11213356; doi:10.1371/journal.pbio.3002678)

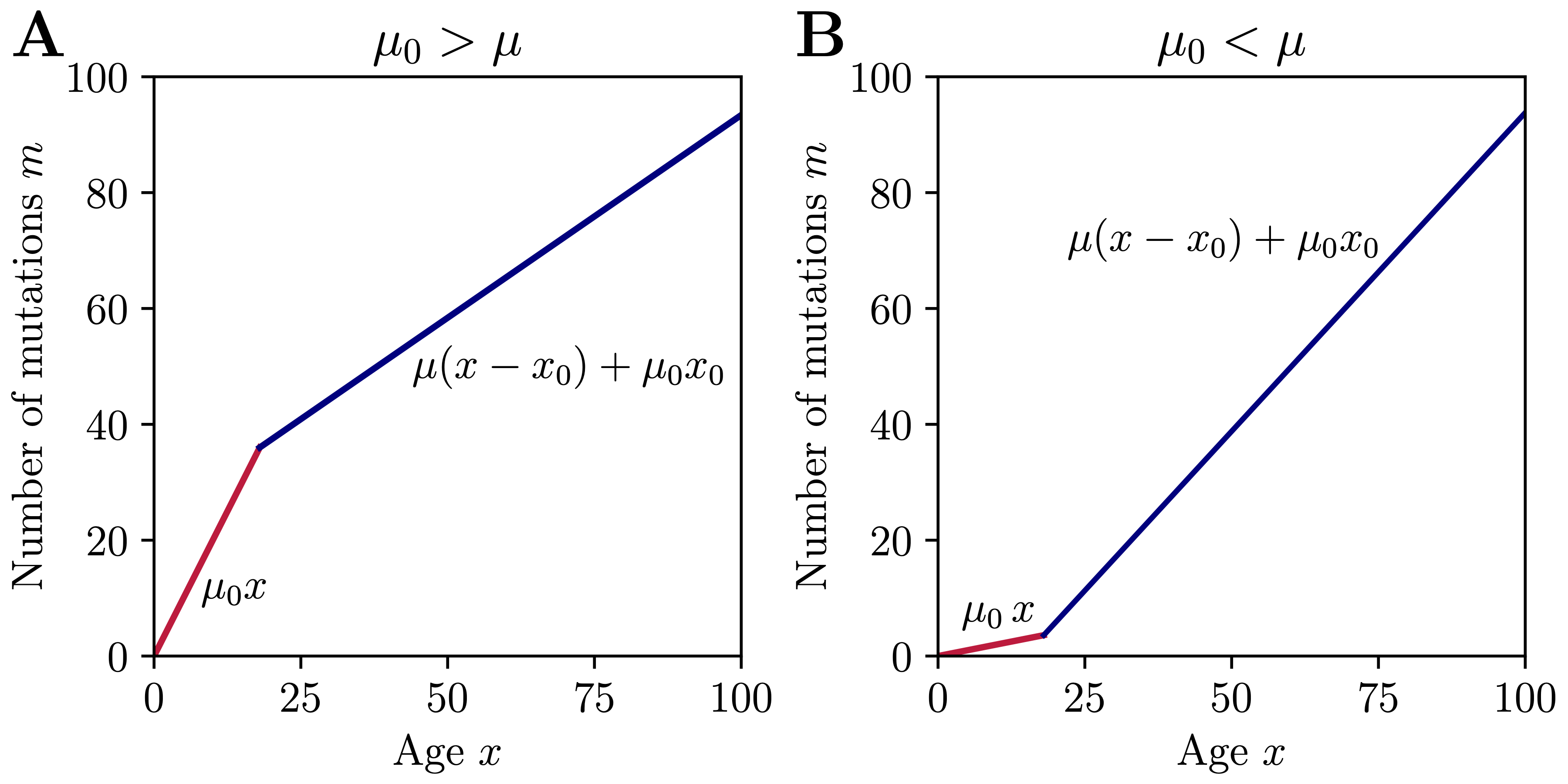

Supplement: S1 Fig — Until age x0 = 18 mutations accumulate at rate μ0 (in red) and after age x0 at rate μ (blue). (A) When μ0>μ, for data from donors of ages x>x0, a regression would yield a positive intercept, b>0. (B) When μ0>μ, such regression would yield a negative intercept, b<0. See discussion in section “Relating model predictions to data” in the main text. Underlying data for this figure can be found in S2 Data. (TIFF) [file pbio.3002678.s001.tiff]

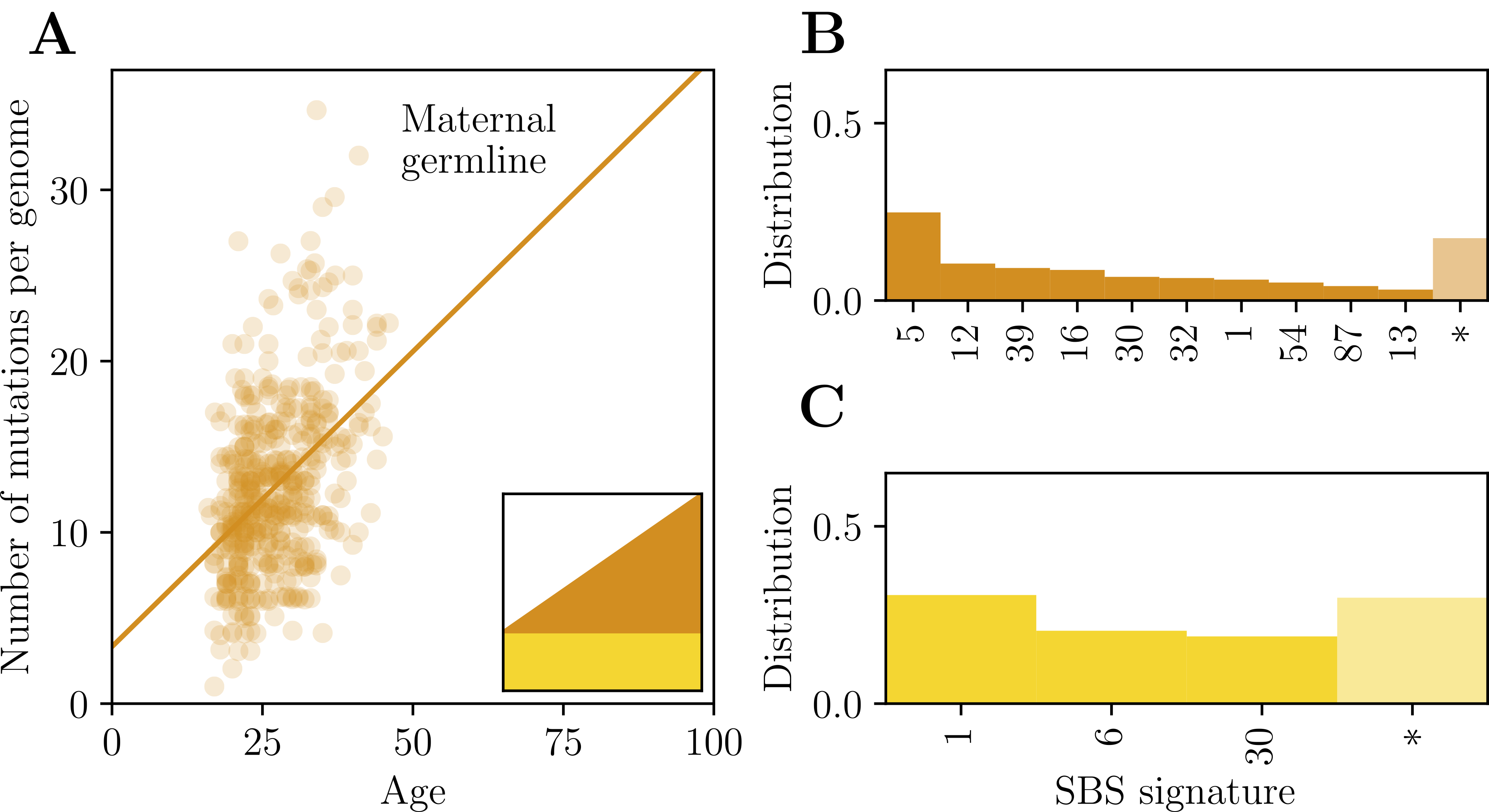

Supplement: S3 Fig — (A) Effect of maternal age on mutations assigned to the maternal germline in pedigree data. Paternal age was included as a covariate in linear regression. (B, C) The decomposition of the maternal mutation spectrum into age-dependent (B) and constant signatures (C); see (A) for the color code. SBS signatures are indicated by their COSMIC label; asterisks indicate unattributed signatures (see Methods). Underlying data for this figure can be found in S2 Data. (TIFF) [file pbio.3002678.s003.tiff]
